# Complement Evasion Strategies of Viruses: An Overview

**DOI:** 10.3389/fmicb.2017.01117

**Published:** 2017-06-16

**Authors:** Palak Agrawal, Renuka Nawadkar, Hina Ojha, Jitendra Kumar, Arvind Sahu

**Affiliations:** Complement Biology Laboratory, National Centre for Cell Science, Savitribai Phule Pune UniversityPune, India

**Keywords:** immune evasion, complement evasion, complement, innate immunity, pathogenesis, DNA viruses, RNA viruses, retroviruses

## Abstract

Being a major first line of immune defense, the complement system keeps a constant vigil against viruses. Its ability to recognize large panoply of viruses and virus-infected cells, and trigger the effector pathways, results in neutralization of viruses and killing of the infected cells. This selection pressure exerted by complement on viruses has made them evolve a multitude of countermeasures. These include targeting the recognition molecules for the avoidance of detection, targeting key enzymes and complexes of the complement pathways like C3 convertases and C5b-9 formation – either by encoding complement regulators or by recruiting membrane-bound and soluble host complement regulators, cleaving complement proteins by encoding protease, and inhibiting the synthesis of complement proteins. Additionally, viruses also exploit the complement system for their own benefit. For example, they use complement receptors as well as membrane regulators for cellular entry as well as their spread. Here, we provide an overview on the complement subversion mechanisms adopted by the members of various viral families including *Poxviridae, Herpesviridae, Adenoviridae, Flaviviridae, Retroviridae, Picornaviridae, Astroviridae, Togaviridae, Orthomyxoviridae* and *Paramyxoviridae*.

## Introduction

The complement system is a constituent of the innate immunity that serves as a vital link between the innate and the adaptive immunity (Carroll and Fearon, 2004; Kolev et al., 2014). This ancient system, established about 1000 million years ago (Nonaka and Kimura, 2006), has the ability to recognize and eliminate varied invading pathogens including viruses. The elimination of viruses by the complement system is owing to direct neutralization of cell-free viruses (Bernet et al., 2003), lysis of the virus-infected cells (Cooper and Nemerow, 1983), induction of antiviral state (Tam et al., 2014), and boosting of virus-specific immune responses due to recognition of effector fragments of complement along with viral antigens by the immune cells (Pyaram et al., 2010b; Kolev et al., 2014; Rattan et al., 2017). Especially, it has been shown that complement promotes the formation of germinal centers and development of antigen-specific antibody responses as a result of co-ligation of B cell receptor and CD21 (a part of CD19-CD21-CD81 complex) by the antigen coupled to C3d (Fearon and Carter, 1995; Fischer et al., 1998) as well as increased retention of such C3d coupled antigens by follicular dendritic cells expressing CD21/CD35 (Fang et al., 1998). In addition, complement has also been shown to enhance virus-specific CD4^+^ and CD8^+^ effector T cells because of signaling through the CRs as well as modulation of antigen-presenting cell function (Kolev et al., 2014). Thus, the complement system exerts a strong selective pressure on viruses, and as a result, functions as a driver of adaptation. In line with this, viruses are known to have developed multiple adaptation strategies against the complement system. In this review article, we discuss how the complement system recognizes and neutralizes various viruses and consequently how viruses subvert the complement assaults.

## Recognition and Neutralization of Viruses by the Complement System

**FIGURE 1:**
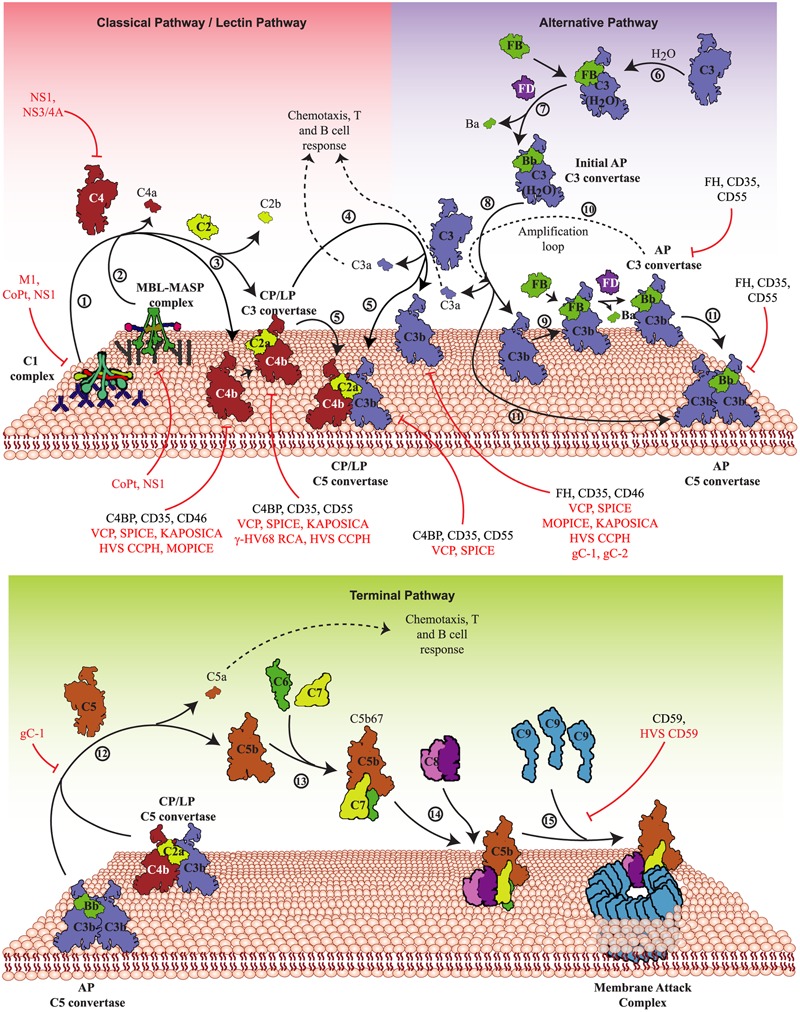
Activation pathways of the complement system and their targeting by viruses. The complement system is activated primarily by three pathways – CP, LP, and AP. **Upper panel**: In the CP, antigen-antibody complexes formed on the pathogen surface are recognized by the C1 complex **(1)** whereas in the LP, specific carbohydrates on the pathogen surface are recognized by MBL/ficolin-MASP complex **(2)**. Both these complexes, upon activation, cleave C4 and C2 that results in the generation of C4bC2a (CP/LP C3 convertase) **(3)**. The CP/LP C3 convertase cleaves C3 into C3b and C3a, where C3b binds and opsonises the pathogen surface and C3a boosts the acquired immune responses **(4)**. When C3b combines with the pre-existing CP/LP C3 convertase, it forms CP/LP C5 convertase **(5)**. In the AP, spontaneous hydrolysis of native C3 by H_2_O (tick-over process) results in the formation of C3b-like C3 [C3(H_2_O)] **(6)**, which binds factor B (FB) and upon cleavage by factor D (FD) forms the initial AP C3 convertase **(7)**. The initial AP C3 convertase then cleaves C3 into C3b and C3a **(8)**. The generated C3b molecules bind to the pathogen surface and initiate the formation of surface-bound AP C3 convertase, C3bBb, with the help of FB and FD **(9)**. The surface-bound AP C3 convertase initiates the AP amplification loop **(10)** resulting in deposition of millions of C3b molecules onto the pathogen surface. Similar to the CP and LP, when C3b combines to the pre-existing AP C3 convertase, it forms the AP C5 convertase **(11)**. **Lower panel**: The C5 convertases cleave C5 into C5b and C5a **(12)**, where C5b binds to C6 and C7 to form a trimer (C5b-7) **(13)** that binds to the pathogen surface, while C5a boosts the acquired immune responses. Further binding of C8 to the trimer results in the formation of C5b-8 that penetrates the membrane **(14)**. Finally, C9 binding to C5b-8 and its polymerization completes the MAC formation leading to lysis **(15)**. These activation pathways are regulated at different steps by host complement regulators like factor H (FH), MCP (CD46) complement receptor-1 (CR-1; CD35), DAF (CD55) and C4b-binding protein (C4BP). Viral proteins that target these pathways are: VCP, SPICE, MOPICE, Kaposi’s sarcoma-associated herpesvirus inhibitor of complement activation (KAPOSICA); γ-HV68 RCA, HVS CCPH; non-structural protein 1 of *Flaviviruses* (NS1); non-structural protein 3/4A of Hepacivirus (NS3/4A); glycoprotein C of HSV-1 (gC-1) and -2 (gC-2), human astrovirus coat protein (CoPt) and M1 protein of INFLV (M1).

The complement system is primarily activated by the recognition of pathogens (or non-self) by the pattern recognition molecules of this system. In addition, the system is also evolved to recognize pathogens without the help of pattern recognition molecules. The three major pathways by which the complement system is activated are the CP, the LP, and the AP (**Figure 1**). The first two pathways are activated with the help of pattern recognition molecules (Degn and Thiel, 2013; Hein and Garred, 2015), while the last pathway is activated spontaneously and without any help from the pattern recognition molecules (Pangburn, 1983). Intriguingly, viruses are known to be recognized by all the three pathways (Rattan et al., 2013).

The CP, LP, and AP converge at the C3 activation step, i.e., cleavage of C3 into C3a and C3b by pathway-specific C3 convertases (C4b,2a and C3b,Bb). The C3b molecules thus generated get covalently attached to the existing C3-convertases resulting in the formation of C5-convertases which cleave C5 into C5a and C5b. Once formed, C5b initiates the formation of C5b-9 or the MAC. The steps involved in the MAC formation include formation of a fluid phase trimer C5b-7, which binds to the membrane. Thereafter, the C8 binds to the membrane attached trimer and initiates binding and polymerization of C9 (i.e., the formation of C5b-9) which gets inserted into the lipid membrane and induces virolysis (**Figure 1**).

The recognition and neutralization of viruses by complement were reported as early as 1930. In this initial study, Douglas and Smith (1930) observed that a heat-labile factor in rabbit serum possesses viricidal activity. Based on the current conception, it can be said that recognition of viral surface by antibodies (IgM, IgG3, and IgG1), C-reactive protein (CRP), serum amyloid P (SAP) or SIGN-R1 (a C-type lectin) and then interaction of these molecules with C1 can lead to activation of the CP resulting in virus neutralization. Most examples studied, however, show that the CP-mediated neutralization requires the presence of antibody (**Table 1**). A few examples nonetheless illustrated the direct interaction of C1q with viral proteins such as gp41 and gp120 of HIV (Ebenbichler et al., 1992; Susal et al., 1994) and p15E of Moloney leukemia virus (Bartholomew et al., 1978).

**Table 1:** Complement-mediated neutralization of various viruses.

Virus families	CP-mediated neutralization	AP-mediated neutralization	LP-mediated neutralization
*Poxviridae*	VACV (Isaacs et al., 1992; Vanderplasschen et al., 1998) EV (Moulton et al., 2008)	EV (Moulton et al., 2008)	**–**

*Herpesviridae*	HSV-1 (Heineman, 1967; Friedman et al., 2000; Hook et al., 2006) HSV-2 (Heineman, 1967; Hook et al., 2006)	EBV (Mold et al., 1988)	HSV-2 (Gadjeva et al., 2004)

*Flaviviridae*	YFV (Schlesinger et al., 1985)	–	DENV (Avirutnan et al., 2010; Fuchs et al., 2010; Thiemmeca et al., 2016) WNV (Avirutnan et al., 2010; Fuchs et al., 2010) YFV (Avirutnan et al., 2010) HCV (Ishii et al., 2001)

*Retroviridae*	HIV-1 (Ebenbichler et al., 1991; Susal et al., 1994; Stoiber et al., 1996) HIV-2 (Ozkaya et al., 2013) HTLV-1 (Ikeda et al., 1998)	–	HIV-1 (Ezekowitz et al., 1989; Saifuddin et al., 2000)

*Togaviridae*	Venezuelan equine encephalitis virus (Brooke et al., 2012)	Sindbis virus (Hirsch et al., 1980b)	–

*Picornaviridae*	–	Coxsackievirus B3 (Anderson et al., 1997)	–

*Orthomyxoviridae*	INFLV A/PR/8/34 (Jayasekera et al., 2007) INFLV A/WS/33 H1N1 (Beebe et al., 1983) INFLV [A(H1N1)pdm09] (Rattan et al., 2017) INFLV A(H3N2)09 (Rattan et al., 2017)	INFLV A(H3N2)09 (Rattan et al., 2017)	INFLV A/Mem71_H_-Bel_N_(H3N1) (Reading et al., 1995)

*Paramyxoviridae*	Parainfluenza type 3 (Vasantha et al., 1988) Newcastle disease virus (Welsh, 1977)	Measles (Sissons et al., 1979, 1980; Devaux et al., 2004) Mumps (Hirsch et al., 1986; Johnson et al., 2008) NiV (Johnson et al., 2011) PIV (Vasantha et al., 1988) Sendai virus (Okada et al., 1979) Simian virus 5 (McSharry et al., 1981; Johnson et al., 2009)	Mumps (Johnson et al., 2008)

Apart from the CP, the LP which is initiated by the recognition of carbohydrate patterns on the pathogen surface by MBL, collectin K-1 (CL-K1) and ficolins (L, H, and M) has also been shown to neutralize viruses (**Table 1**). In case of the LP, however, only MBL has been shown to recognize and neutralize viruses, e.g., oncolytic viruses (Wakimoto et al., 2002), HCV (Ishii et al., 2001), and INFLV (Thielens et al., 2002) (**Table 1**). The AP, which is activated spontaneously, is capable of neutralizing viruses directly as well as owing to its activation by the CP and LP [i.e., due to activation of the AP loop by CP and LP (Moulton et al., 2008; Rattan et al., 2017)]. The examples include EBV, Sindbis virus, MuV, MeV, etc. (**Table 1**). For the AP-mediated complement neutralization though, it is important that the viral surface is amenable to C3b deposition as this is a key step for activation of this pathway.

## Modes of Complement-Mediated Neutralization of Viruses

Neutralization of viruses refers to the loss of viral infectivity due to binding of antibody and/or complement to the viral surface. Neutralization of viruses by complement occurs owing to different mechanisms. The first mechanism identified for complement-mediated neutralization of viral infectivity was virolysis. Berry and Almeida (1968) showed that incubation of avian infectious bronchitis virus with antibody and complement produced holes of ∼100 Å diameter in the virus membrane. More reports in the subsequent years showed that the terminal components of complement form a MAC on the viral envelopes, resulting in the holes and thereby virolysis (Cooper, 1998). The examples include alphaviruses, herpesviruses, coronaviruses, retroviruses, and paramyxoviruses [reviewed in (Cooper and Nemerow, 1983)]. However, virolysis is not the only mechanism by which complement neutralizes viruses. Soon after virolysis was suggested as a mechanism of virus neutralization, Daniels et al. (1970) showed that HSV can be neutralized by adding only the early complement components. This then raised a question: How many components are necessary for virus neutralization? Studies performed in the following years, until now, showed that the requirement of early complement components for neutralization vary from virus to virus. For instance, direct binding with C1q and MBL is enough to neutralize HTLV-1 (Ikeda et al., 1998) and SARS coronavirus (Ip et al., 2005), respectively, while deposition of components up to C3b is necessary for neutralization of WNV (Fuchs et al., 2010), DENV (Avirutnan et al., 2011a), EV (Moulton et al., 2008), INFLV (Jayasekera et al., 2007), and VSV (Beebe and Cooper, 1981). In the case of HSV-1 gC null virus, in particular, deposition of components up to C5 was shown as necessary for neutralization (Friedman et al., 2000). In most cases, however, it is not clear if such depositions affect the attachment and entry of virions or post entry processes. Intriguingly, apart from neutralization of viral infectivity, a recent study has reported that non-enveloped viruses coated with C3b are capable of triggering antiviral signaling as well as targeting of these virions for rapid proteasomal degradation (Tam et al., 2014).

The other two modes of neutralization are aggregation and phagocytosis. Opsonization of viral surface with complement components can lead to aggregation of viruses as well as phagocytosis of these viruses via CRs present on the phagocytic cells. An aggregation-mediated neutralization of viruses, which is because of decrease in the total number of infectious virus units, was observed in polyoma virus (Oldstone et al., 1974), INFLV (Jayasekera et al., 2007), and simian virus (Johnson et al., 2008), whereas phagocytosis-mediated virus neutralization was reported for HSV (Van Strijp et al., 1989) and JEV (Van Strijp et al., 1990).

## Viral Strategies for Targeting Complement

### Overview

**FIGURE 2:**
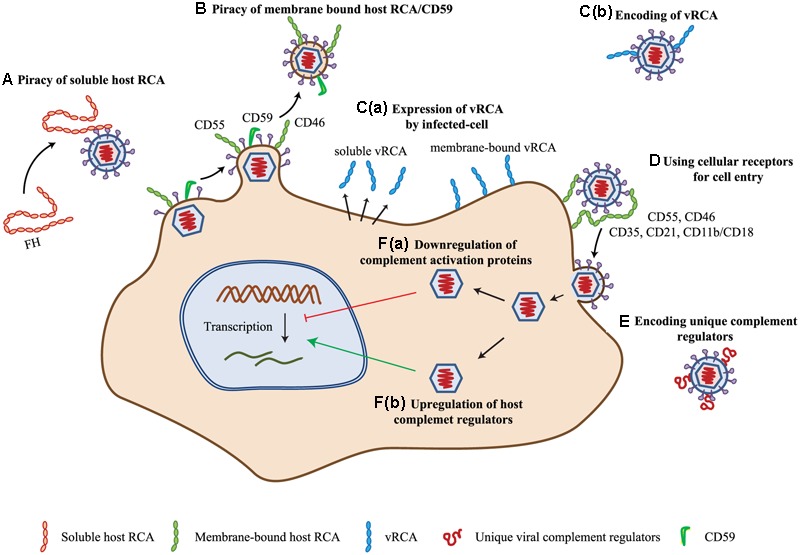
Complement evasion strategies of viruses. **(A)** Piracy of soluble host RCA. Viruses evade complement attack by recruiting soluble complement regulator, viz. complement factor H (FH) by members of the families *Flaviviridae, Retroviridae*, and *Togaviridae*. **(B)** Piracy of membrane-bound host RCA. During budding, many enveloped viruses (viz. members of *Poxviridae, Herpesviridae, Flaviviridae, Retroviridae, Orthomyxoviridae*, and *Paramyxoviridae*) recruit membrane-bound regulators like CD55, CD46, and CD59. **(C)** Encoding homologs of RCA (vRCA). Viruses belonging to the families *Poxviridae* and *Herpesviridae* have been shown to encode regulators which are homologs of the human RCA gene family proteins. These are expressed as soluble **[C(a)]** as well as membrane-bound **[C(b)]** proteins. **(D)** Use of complement regulators and receptors for cellular entry. Viruses of the families *Herpesviridae, Adenoviridae, Flaviviridae, Retroviridae, Picornaviridae*, and *Paramyxoviridae* are known to use complement receptors and regulators for cellular entry (e.g., CD35, CD21, CD11b/CD18, CD55, and CD46). **(E)** Encoding of unique complement regulatory proteins. Apart from vRCA, members of some virus families namely, *Herpesviridae, Flaviviridae*, and *Astroviridae* encode unique complement regulatory proteins for evading the complement system. **(F)** Modulation of complement protein expression. Viruses are also known to modulate complement proteins for their benefit. These include down-regulation of complement activation proteins **[F(a)]** and up-regulation of complement regulatory proteins **[F(b)]**. Members of *Herpesviridae, Flaviviridae*, and *Paramyxoviridae* are involved in up-regulation of host complement regulators, while that of *Flaviviridae* are known to down-regulate the expression of complement activation proteins. Key: CD55, decay-accelerating factor; CD46, membrane cofactor protein; vRCA, viral regulators of complement activation; CD35, CD21, CD11b/CD18, complement receptor-1, -2 and -3.

The uncovering of viral strategies for targeting the complement system started as early as 1976 when Jondal et al. (1976) reported that EBV receptor on the human B cell surface is related to the receptors for complement C3. Later, a more definitive report demonstrated that it is the CR-2 (CD21) that serves as an entry receptor for EBV on the human B lymphocytes (Fingeroth et al., 1984). This strategy of using CRs for cellular entry has been shown to be employed by many viruses wherein apart from CR-2, CRs like CR-3 (CD11b/CD18) and CR-1 (CD35) were also shown to be the targets of viruses (Ogembo et al., 2013; Posch et al., 2015) (**Figure 2D**). In addition to the receptors, complement regulators viz. DAF (CD55) and MCP (CD46) have also been shown to be utilized for cellular entry by viruses (**Figure 2D**) (see sections below).

In enveloped viruses, the content of the envelope is influenced by the host as they are derived from the host cell membranes. Because the AP activation was shown to be influenced by the quantity of sialic acid present on the activating surface owing to the recruitment of factor H (Fearon, 1978), efforts were made to determine whether host modified sialic acid content of virus influences its clearance. Hirsch et al. (1981, 1983) showed that variation in the sialic acid content of Sindbis virus indeed influences its clearance from the blood, which is dependent on complement. The host membranes, however, are decorated with complement regulators, which suggested a possibility that incorporation of these in the viral envelopes is likely to protect them from the complement assault. A formal demonstration of this supposition came from the study of Saifuddin et al. (1995) who established that incorporation of the complement regulators CD55 and CD59 in human immunodeficiency virus (HIV) envelope provide protection against complement. Later, this strategy was shown to be utilized by several enveloped viruses (see sections below) (**Figures 2A,B**).

Besides recruitment of host complement regulators, viruses have also been shown to encode their own complement regulators for protection from the host complement. The discoveries of these regulators, however, were accidental. The first virally encoded complement regulator identified was gC of HSV-1 (Friedman et al., 1984). Originally, it was identified as a C3b receptor, but subsequently, it was shown to inhibit complement activation (Friedman et al., 1984; Fries et al., 1986) (**Figure 2E**). Similarly, the first identification of a homolog of human RCA gene family proteins, encoded by VACV, was spearheaded by the observation that an attenuated strain of VACV (designated as 6/2) does not encode this protein (Kotwal and Moss, 1988; Kotwal et al., 1990). Hereafter, multiple laboratories ardently started looking for the presence of such regulators in viruses and were able to find these only in large DNA viruses, namely poxviruses and gammaherpesviruses (**Figure 2C**) (see Section “Complement Subversion by Poxviruses” and “Complement Subversion by Herpesviruses”).

Betaherpesviruses do not encode complement regulators, yet cells infected by its family member HCMV was shown to persist in the presence of antibodies to the virus. Spiller et al. (1996) thus hypothesized that HCMV infection may modulate the expression of cell surface complement regulators. Experimental verification of this premise by them indeed showed that complement regulators CD46 and CD55 are up-regulated on the infected cells and are responsible for averting complement activation on these cells [**Figure 2F(b)**]. Viruses seem to have not only been involved in up-regulation of complement regulators, but also in down-regulation of complement components essential for complement activation. One such example is HCV. Banerjee et al. (2011), Mazumdar et al. (2012), and Kim et al. (2013, 2014) showed that HCV suppresses the expression of C2, C4, C3, and C9, which in turn can result in inhibition of the CP/LP C3 convertase formation [**Figure 2F(a)**].

### Complement Subversion by Poxviruses

Poxviruses are large brick-shaped (240 nm by 300 nm) enveloped viruses that contain linear double-stranded DNA genomes (mostly 180–290 kbp) forming a covalently closed hairpin loop at the terminus (Buller and Palumbo, 1991). The poxvirus family is divided into two subfamilies – *Chordopoxvirinae* (poxviruses that infect vertebrates) and *Entomopoxvirinae* (poxviruses that infect insects). The two most important members of *Chordopoxvirinae* are variola virus that causes smallpox, and VACV that was used to control smallpox. Both these viruses have been shown to efficiently evade the complement system.

Complement evasion in poxviruses was first identified in VACV. It was shown that one of the two major secretory protein of VACV is a structural homolog of the RCA proteins (Kotwal and Moss, 1988) and possesses complement regulatory activity (Kotwal et al., 1990; Mckenzie et al., 1992; Sahu et al., 1998). This four CCP domain containing protein was dubbed as VCP. Initial functional characterization of the protein showed that it has the ability to bind to complement proteins C3b and C4b and accelerate the decay of CP/LP as well as AP C3 convertases (termed decay-accelerating activity) (Mckenzie et al., 1992). Later, it was also shown to support the inactivation of C3b and C4b with the help of factor I (termed cofactor activity) (Sahu et al., 1998). The importance of this protein was further enhanced by Isaacs et al. (1992) study which showed that VCP plays a role in the pathogenesis by avoiding the antibody-mediated neutralization of virus which is complement dependent. VCP was also shown to interact with heparan sulfate proteoglycans and A56 viral protein which impart surface binding property to the protein (Smith et al., 2000; Girgis et al., 2008; Dehaven et al., 2010). Thus, VCP possesses the ability to inhibit complement in solution as well as on the cell/viral surface.

Variola is a human-specific virus, which encodes an ortholog of VCP. Rosengard et al. (2002) characterized this protein to investigate if it is more potent than VCP in inhibiting human complement. The protein was found to be 100-fold more potent in inactivating C3b and 6-fold more potent in inactivating C4b (Rosengard et al., 2002). The protein was named as SPICE (Rosengard et al., 2002). Because SPICE differs from VCP in only 11 amino acids, subsequent efforts were directed to determine the hotspots in SPICE responsible for this substantial increase in activity and it was credited to four amino acids (Sfyroera et al., 2005; Yadav et al., 2008). VACV is feral in India and Brazil and is known to cause repeated outbreaks in dairy cattle. When VCP and SPICE were examined for their ability to inhibit bovine complement, it became apparent that VCP is highly efficient (33-fold) than SPICE in inhibiting bovine complement and intriguingly this was due to the charge reversal in the central domains of VCP (Yadav et al., 2012).

Among the MPXV, the Congo Basin strains were shown to encode VCP orthologs (named MOPICE). *In vitro* studies demonstrated that it possesses cofactor activity for C3b and C4b, but lacks decay-accelerating activity (Liszewski et al., 2006). Interestingly, MOPICE was found to be absent in the less virulent West African strains of MPXV (Chen et al., 2005; Likos et al., 2005). Consequently, investigations were performed to determine whether the low virulence of West African strains was due to the absence of MOPICE. An *in vivo* study was performed utilizing two recombinant MPXVs: the West African strain incorporated with MOPICE and the Congo Basin strain devoid of MOPICE. Incorporation of MOPICE in the West African strain showed some changes in the disease manifestations, but not in disease-associated mortality. Removal of MOPICE from the Congo Basin strain, however, reduced disease morbidity and mortality without affecting the viral load. Together the results suggested that though the difference in virulence in these two strains is not due to MOPICE, the protein does play a moderate role in pathogenesis (Hudson et al., 2012). Apart from the above-mentioned poxviruses, functional complement regulator was also found in cowpox virus [named IMP (Miller et al., 1997)] and EV [named EMICE (Moulton et al., 2010)].

Another immune evasion strategy used by VACV is by acquiring host complement regulators into its envelope. Two distinct types of virions are produced by VACV: intracellular mature virus (IMV) and extracellular enveloped virus (EEV). Of these virions, only EEV is resistant to complement-mediated neutralization in the absence of specific antibodies. Vanderplasschen et al. (1998) demonstrated that the resistance to complement in EEV is due to the incorporation of the host complement regulators CD46, CD55, and CD59 into the outer envelope of EEV. The relative role of each of these regulators in protection against complement nonetheless is not yet clear.

### Complement Subversion by Herpesviruses

Like poxviruses, herpesviruses are also large (120–260 nm) enveloped DNA viruses with linear double-stranded genomes of about 124–230 kbp (Liu and Zhou, 2007). Remarkably, a large part of their genomes encodes for genes that are involved in host control. They are classified into three subfamilies – *Alphaherpesvirinae, Betaherpesvirinae*, and *Gammaherpesvirinae* – and all the subfamilies are known to subvert the complement system.

#### Alphaherpesviruses

The members of *Alphaherpesvirinae* encode gC that target complement. The gCs of HSV type 1 (gC-1) as well as type 2 (gC-2) have been studied very well. It is clear that deletion of gC-1 and gC-2 from the respective viruses result in their effective neutralization by complement (Friedman et al., 1984; Lubinski et al., 1999). Data obtained up until now suggest that interaction of gC-1 and gC-2 with C3b helps them evade the complement system.

Direct binding experiments have shown that both gC-1 and gC-2 bind to C3 and its activation products (C3b, iC3b, and C3c), but not C4b (Fries et al., 1986; Harris et al., 1990; Kostavasili et al., 1997; Rux et al., 2002). Further, it has been shown that interaction of gC-1 with C3b blocks its interaction with properdin and C5 (Harris et al., 1990; Kostavasili et al., 1997) resulting in inhibition of complement activation. The importance of gC-1 domains that interact with C3b and that blocks properdin/C5 interaction with C3b has also been examined *in vivo* and both the domains have been shown to contribute to virulence (Lubinski et al., 1999). Of note, the domain that blocks properdin and C5 interaction with C3b is absent in gC-2 suggesting that its mechanism of complement subversion is different than that of gC-1 (Kostavasili et al., 1997).

Other than gCs, HSV-1 also encodes glycoproteins gE and gI, which together form a heterodimeric complex (Johnson and Feenstra, 1987; Johnson et al., 1988) and function as IgG Fc receptor (FcγR) (Dubin et al., 1991; Lubinski et al., 2011). It was proposed that FcγR participates in immune evasion by promoting ‘antibody bipolar bridging,’ i.e., HSV antibody interacts with the FcγR on one end and target antigen on the other resulting in inhibition of Fc domain functions (Frank and Friedman, 1989). This notion was well-supported by the crystal structure of HSV-1 FcγR bound to IgG Fc (Sprague et al., 2006). In addition, *in vitro* studies showed that presence of FcγR protects HSV-1 from antibody-dependent complement-mediated neutralization and antibody-dependent cellular cytotoxicity (Frank and Friedman, 1989; Dubin et al., 1991; Lubinski et al., 2011). These results were also corroborated by *in vivo* infection studies (Lubinski et al., 2011). Together, the aforementioned studies provided compelling evidence that immune evasion molecules such as gC and gE/gI play an essential role in HSV infection. These results also prompted the formulation of a trivalent subunit antigen vaccine for genital herpes which contains gC-2 and gE-2 apart from gD-2 that is essential for viral entry (Awasthi et al., 2014).

#### Betaherpesviruses

Viruses belonging to this family have been shown to protect themselves from host complement by acquiring complement regulators from the host cells as well as by up-regulating the expression of complement regulators on the infected cells. In addition, these viruses have also been demonstrated to utilize CD46 as a cellular receptor.

In particular, during maturation, HCMV was shown to acquire complement regulators like CD55 and CD59 from the host cells. Further, treatment of the virions with antibodies against the acquired regulators showed a reduction in the infectious titer when incubated with complement indicating that the acquired regulators help to protect the virus from complement (Spear et al., 1995). Apart from gaining the regulators from the host cell surface during budding, HCMV (and MCMV) also up-regulates the expression of complement regulators such as CD55 and CD46 on the infected cells (Spiller et al., 1996; Nomura et al., 2002). Other than HCMV, HHV-7 was also demonstrated to up-regulate the expression of CD59 and CD46 on the infected cells during the late stage of infection (Takemoto et al., 2007).

Human herpesvirus-6 is a collective term used for two ubiquitous human betaherpesviruses – HHV-6A and HHV-6B. Though these viruses have a broad tropism for different human cell types, only a restricted number of non-human primate species show susceptibility to HHV-6. Thus, efforts were directed at identifying a receptor that is ubiquitous, yet have significant species differences. Intriguingly, this search led to the identification of CD46 as a cellular receptor for HHV-6 (Santoro et al., 1999). The extracellular region of CD46 contains four CCP domains. Identification of CD46 domains crucial for interacting with the virions revealed that only CCP domains 2 and 3 are essential for the interaction (Greenstone et al., 2002). Initial attempts to ascertain the viral glycoprotein that interacts with CD46 resulted in the identification of glycoprotein H (gH) as the ligand. Later investigations, however, demonstrated that heterotetramer gH/gL/gQ1/gQ2 is a viral ligand for CD46 (Mori et al., 2004; Kawabata et al., 2011; Tang et al., 2011). More recently, it has been demonstrated that apart from the heterotetramer complex, gB is also essential for the membrane fusion by HHV-6 (Tanaka et al., 2013). Among the components of the heterotetramer, gQ1 and gQ2 seem to play a critical role in binding to CD46 (Jasirwan et al., 2014).

#### Gammaherpesviruses

Gammaherpesviruses have been shown to encode homologs of the human RCA and CD59 that help them elude the complement system. Moreover, they also utilize CRs for cellular entry, which are widely expressed on the host cells.

The presence of a functional homolog of the human RCA was first observed in HVS whose natural host is a squirrel monkey (*Saimiri sciureus*) (Falk et al., 1972). Albrecht and Fleckenstein (1992) showed that HVS *ORF4* encodes a four CCP domain containing the RCA homolog, which is expressed as a soluble (named sCCPH) as well as membrane-anchored (named mCCPH) protein. Initial characterization of this protein showed that it has the ability to inhibit complement activation (Fodor et al., 1995). Later, its detailed functional characterization showed that like poxviral RCA proteins, it also has the ability to accelerate decay of the CP/LP C3 convertase and to a lesser extent the AP C3 convertases as well as inactivate C3b and C4b resulting in inhibition of the CP/LP as well as the AP (Singh et al., 2006). Besides, the domain requirement of this protein for complement inactivation was essentially similar to that of the poxviral RCA proteins (Singh et al., 2009; Reza et al., 2013). Unlike any other virus, HVS also encodes a homolog of the terminal complement inhibitor CD59 (Albrecht et al., 1992). Expression of HVSCD59 on the surface of different cells was shown to protect these cells from the complement-mediated damage (Rother et al., 1994; Bramley et al., 1997).

Similar to HVS, KSHV (HHV-8) *ORF4* also encodes an RCA homolog (Russo et al., 1996). The KSHV RCA homolog was characterized simultaneously by two laboratories and named as Kaposica (Mullick et al., 2003) and KCP (Spiller et al., 2003a). Structurally, the full-length Kaposica is composed of four CCP domains, a dicysteine motif, a serine/threonine (S/T) rich region and a transmembrane domain (Russo et al., 1996). Its spliced variants, on the other hand, either lack the S/T region or the dicysteine motif and the S/T region (Spiller et al., 2003b). Importantly, it is expressed on the infected cells as well as on the virion envelope (Spiller et al., 2006). The protein was shown to function at the level of C3 convertases – it efficiently decays the CP/LP C3 convertase with little activity against the AP C3 convertase, and supports inactivation of C3b and C4b with the help of factor I (Mullick et al., 2003, 2005). In-depth mapping of its functional domains and mutational analysis revealed that it functions in a manner similar to that of the human RCA proteins (Mullick et al., 2005). Electrostatic modeling and mutagenesis studies indicated that the positive electrostatic potential in domains 1 and 4, and in the linkers between domains 1–2 and 2–3, influence the complement regulatory activities of Kaposica (Pyaram et al., 2010a). More recently, efforts were made to understand the molecular mechanism behind its cofactor activity. The data revealed that apart from its interaction with factor I, bridging of the CUB and MG2 domains of C3b/C4b by Kaposica is critical for imparting cofactor activity (Gautam et al., 2015). This model also seems to be true for the human RCA proteins (Gautam et al., 2015).

Murine gammaherpesvirus 68 also encodes a four CCP domain homolog of RCA that exist in both soluble and membrane-bound forms (Kapadia et al., 1999). The γ-HV68 RCA was shown to inhibit C3b deposition on the complement activating particle zymosan (Kapadia et al., 1999) suggesting that it acts at the C3 convertase level, however, the mechanistic details of its regulation are not clear. The protein was also examined for its role during the infection which suggested that it is vital for viral pathogenesis (Kapadia et al., 2002). Specifically, it was shown that the γ-HV68 RCA-null virus is attenuated in the WT mice, but not in the C3^-/-^ mice suggesting that its interaction with C3 is important for *in vivo* pathogenesis (Kapadia et al., 2002). Another member of *Gammaherpesvirinae* that encode RCA homolog is RRV. Two different strains of RRV (H26-95 and 17577) have been shown to encode RCA homologs which differ in the structure due to the varied CCP content. Both have been shown to display the complement regulatory functions (Okroj et al., 2009).

Unlike the above-mentioned viruses, EBV targets the complement system for its entry into B cells. We now know that it targets CR-1 (Ogembo et al., 2013) as well as CR-2 (Fingeroth et al., 1984; Nemerow et al., 1985; Tanner et al., 1987) for its cellular entry. Its entry using CD35, however, requires the co-expression of HLA Class II molecules (Ogembo et al., 2013). The EBV protein that specifically interacts with CD21 is gp350/220 [*K*_D_ = 1.2 × 10^-8^ M (Tanner et al., 1988)]. Mapping of CD21 interaction regions in gp350/220 indicated that the N-terminal 470 amino acid is essential for binding to CD21 (Tanner et al., 1988). These results were also corroborated by the X-ray structure of the gp350 (Szakonyi et al., 2006) and other experiments wherein an EBV-neutralizing monoclonal antibody 72A1 directed against the N-terminal epitope was shown to restrict the entry of EBV, and the gp350/220 mutant lacking N-terminal amino acids was shown to have negligible ligand activity (Tanner et al., 1988).

### Complement Subversion by Adenoviruses

Adenoviruses are medium sized (90–100 nm) non-enveloped DNA viruses that contain a linear double-stranded genome of 26–48 kbp (Wold and Horwitz, 2007; Arnberg, 2012). They are classified into five genera of which human AdV of the genus *Mastadenovirus* have been widely studied and divided into seven species (A to G) which contain more than 50 serotypes. Certain serotypes of human AdV have been shown to target CD46 for their cellular entry.

Human AdVs primarily utilize CAR for their attachment and infection (Bergelson et al., 1997). However, group B serotypes AdV-11 and AdV-35 and group D serotype AdV-37 have been demonstrated to utilize CD46 as a cellular attachment receptor (Gaggar et al., 2003; Segerman et al., 2003; Wu et al., 2004). It was also shown that the fiber knob protein of these viruses helps them to interact with the extracellular domain of CD46. Moreover, the investigations also revealed that expression of CD46 on the non-permissive cells renders them susceptible to viral attachment and infection (Segerman et al., 2003). Efforts to decipher the mechanism of molecular recognition between CD46 and fiber knob revealed that this interaction requires contact between two N-terminal CCP domains of CD46 with two fiber knob monomers (Persson et al., 2007; Wang et al., 2007; Pache et al., 2008). CD46 is largely expressed as four isoforms (BC1, BC2, C1, and C2) owing to alternative splicing of the coding regions for serine/threonine/proline (STP)-rich domains (B and C) and the cytoplasmic tails (1 and 2). Consequently, a study in particular with AdV-37, also looked at the utilization of various CD46 isoforms for infection by this serotype. The data revealed that AdV-37 utilized the C2 isoform of CD46 as a cellular receptor (Wu et al., 2004).

### Complement Subversion by Flaviviruses

*Flaviviruses* (members of the family *Flaviviridae*) are enveloped, roughly spherical (∼50 nm), single-stranded, positive-sense RNA viruses with genome of ∼11 kb, which codes for three structural [capsid (C), membrane (prM/M), and envelope (E)] and seven non-structural (NS) proteins (NS1, NS2A, NS2B, NS3, NS4A, NS4B, and NS5) (Simmonds et al., 2017). One of the NS proteins, NS1, has been shown to function as a complement regulator. The important human pathogens belonging to this genus include YFV, DENV, WNV, JEV, and ZIKV.

Non-structural protein 1 is a 48 kDa glycoprotein of the flaviviruses which is required for effective viral replication (Mackenzie et al., 1996). Remarkably, the protein has the capability to subvert all the three major activation pathways of complement. It is synthesized as a soluble monomer that dimerizes upon post-translational modification in the endoplasmic reticulum. After its secretion from the infected cells, it forms large oligomers, e.g., hexamers. Early studies on its interaction with the complement system described it as a “soluble complement fixing antigen” (Brandt et al., 1970). However, later it became clear that NS1-mediated activation of complement was due to the formation of NS1-antibody immune complexes (Avirutnan et al., 2006). We now know that NS1 directly binds and recruits factor H (Chung et al., 2006), C4b binding protein (C4BP) (Avirutnan et al., 2011b), clusterin (Kurosu et al., 2007), and vitronectin (Conde et al., 2016) to the surface of infected cells resulting in inhibition of the AP, CP, and LP, and MAC formation. Interestingly, NS1 has also been shown to directly interact with C9 and inhibit its polymerization (Conde et al., 2016). A comparison of the activity revealed that NS1 from ZIKV is more potent than NS1 from DENV2 and WNV (Conde et al., 2016). Further, the soluble hexameric NS1 is also known to interact with C4 and pro-C1s forming a trimolecular complex C4-NS1-C1s which promotes the cleavage of C4 to C4b consuming the molecule in solution (Avirutnan et al., 2010). A recent report has also established that NS1 competitively binds to MBL which prevents the later from recognizing and neutralizing DENV (Thiemmeca et al., 2016). Thus, multiple interactions of NS1 with the complement components and regulators seem to enhance its effectiveness as a complement regulator.

### Complement Subversion by Hepaciviruses

*Hepaciviruses* (members of the family *Flaviviridae)* contain single-stranded positive-sense RNA genome of about 9.6 kb. Among these viruses, HCV is the major human pathogen that causes hepatitis, hepatocellular carcinoma and lymphoma (Sanyal et al., 2010; Tasleem and Sood, 2015). It codes for 10 proteins: the structural core, E1, E2 and non-structural NS1, NS2, NS3, NS4A, NS4B, NS5A, NS5B (Simmonds et al., 2017). Studies performed to understand the complement evasion mechanisms of HCV suggest that the virus utilizes three distinct strategies to escape the complement-mediated attack.

The first strategy utilized by the virus is cleavage of the CP/LP component C4, which is unique to HCV. Examination of the effect of various HCV proteins on the complement component C4 showed that NS3/4A protease can cleave the γ-chain of the molecule resulting in inhibition of complement activation (Mawatari et al., 2013). Importantly, such cleavage of C4 was also observed in HCV-infected cells expressing C4. The second strategy involves incorporation of host complement regulators onto its envelope. Like other enveloped viruses, HCV was also shown to incorporate CD59 (Amet et al., 2012) as well as CD55 (Mazumdar et al., 2013) into its envelope for protection against complement-mediated neutralization. The virus as well as the core protein was also shown to up-regulate the expression of CD55 onto the hepatocytes (Mazumdar et al., 2013; Kwon et al., 2016). The third strategy that HCV employs is regulation of complement synthesis. The virus effectively inhibits the expression of C2 (Kim et al., 2014), C4 (Banerjee et al., 2011), C3 (Mazumdar et al., 2012), and C9 (Kim et al., 2013) and thus inhibits the CP/LP C3 convertase formation and MAC formation.

Apart from this, the HCV core protein was also shown to interact with the CR gC1qR and inhibit the proliferation of T cell (Yao et al., 2001b), owing to reduction in IL-2 and IL-2R gene transcription (Yao et al., 2001a). Additionally, the core protein was also shown to reduce the secretion of IL-12 by monocytes/macrophages (Zhang et al., 2011) and expression of the MHC-I on dendritic cells (O’Beirne et al., 2009) suggesting that it mediates immune dysregulation by interacting with gC1qR.

### Complement Subversion by Retroviruses

Retroviruses are spherical (100 nm in diameter), enveloped, single-stranded, positive strand RNA viruses with a DNA intermediate. The virus contains two identical copies of genomic RNA of 7–10 kb (Coffin, 1992). The family *Retroviridae* is divided into subfamily *Orthoretrovirinae* and *Spumaretrovirinae*. HIV-1, which falls under the genus Lentivirus of *Orthoretrovirinae* subfamily, has been well-studied for its interaction with complement. It maintains an intricate balance of complement activation and regulation to achieve maximum infection without undergoing lysis (Stoiber et al., 2008).

During HIV infection, a significant number of free virions can be found in the plasma of infected person suggesting these virions must be resistant to complement-mediated virolysis (Ho et al., 1995; Wei et al., 1995). Saifuddin et al. (1995) therefore looked for the incorporation of host complement regulators into the virions and observed that HIV obtains glycosylphosphatidylinositol (GPI)-anchored proteins CD55 and CD59 on its surface which then provide resistance against complement-mediated lysis. In addition, HIV was also shown to acquire the complement regulator CD46 while budding (Saifuddin et al., 1997). Examination of the role of individual complement regulators in virion protection from complement revealed that each of the regulators plays a protective role (Saifuddin et al., 1997). Since the expression levels of CD55, CD46, and CD59 vary among different cell types, the sensitivity or resistance of HIV to complement depends on the type of host cell from which the virions are derived (Saifuddin et al., 1997). Similar to HIV, SIV and HTLV-1 also acquire host cell-derived membrane complement regulators onto their surface (Montefiori et al., 1994; Spear et al., 1995). Factor H is another complement regulator which is abundantly present in the body fluids. HIV has also been demonstrated to recruit factor H on its surface via interaction with gp41 and gp120 (Pinter et al., 1995a,b) providing yet another efficient mechanism to inhibit complement, in particular, the AP. An illustration of this premise was performed using an *in vitro* experiment wherein HIV virions were shown to be lysed in a complement-dependent fashion when treated with factor H-deficient sera and anti-HIV antibody (Stoiber et al., 1996).

Human immunodeficiency virus is well-known to employ complement for its benefit. The first observation was made by Robinson et al. (1988, 1989) who demonstrated using patient serum that liaison between antibody and complement results in enhanced HIV infection. This phenomenon was later dubbed as complement-mediated antibody-dependent enhancement of HIV infection (C-ADE) (Robinson et al., 1990). Afterward, multiple studies including experimental infection in rhesus macaques (*Macaca mulatta*) with SIV presented data supporting this thesis [reviewed in detail in (Stoiber et al., 2008)]. The complement opsonized virions bound to CD35 on erythrocytes are also liable for spread to antigen-presenting cells bearing CR-2, -3, and -4 (Hess et al., 2002). Specifically, it was hypothesized that CD35 on erythrocytes can support the cleavage of C3b attached to HIV with the help of factor I, the cleaved C3b (iC3b or C3dg) has less affinity for CD35 and hence will be transferred to antigen-presenting cells decorated with receptors for inactivated C3b. Supporting this, iC3b coated HIV display enhanced infection of monocytes/macrophages and DCs expressing CR-3 and CR-4, wherein the complement-opsonized HIV efficiently bypasses the SAMHD1 restriction in DCs (Posch et al., 2015). Similarly, C3d coated HIV were shown to be associated with B cells that express CD21 (Moir et al., 2000). It is likely that these viruses carrying B cells disseminate infection systemically. Further, *in vitro* experiments have also shown the CD21 dependent transfer of HIV from B cells to T cells (Jakubik et al., 2000; Moir et al., 2000). In addition to B cells, follicular dendritic cells also trap HIV which is maintained in the germinal centers (Joling et al., 1993; Kacani et al., 2000) and it is believed that this is critical for the pathogenesis of HIV infection (Stoiber et al., 2003).

### Complement Subversion by Picornaviruses

Picornaviruses are small, spherical (∼30 nm), non-enveloped, positive-sense single-stranded RNA viruses with a genome size of about 7.5 kb (Jiang et al., 2014). The echoviruses, coxsackieviruses, and enteroviruses belonging to the genera enterovirus have been shown to employ CD55 as a cellular receptor or as a binding protein for attachment.

The first observation that echoviruses (namely echo-6, -7, -11, -12, -20, and -21) use CD55 as a receptor for attachment and infection was made by Bergelson et al. (1994). The evidence provided include: (i) loss of the capacity to bind to echo-7 by HeLa cells after treatment with phosphatidylinositol-specific phospholipase C, which removes GPI-anchored proteins from the cell surface, (ii) binding of echo-7 to CHO cells transfected with CD55, (iii) prevention of echovirus infection in HeLa cells by anti-CD55 antibody. Experimentations sought to determine CD55 domains critical for the echo-7 binding established the requirement of CCPs 2-4 (Clarkson et al., 1995). In echo-11 and -12, however, CCP3 of CD55 was identified to be primarily important for the contact (Williams et al., 2003). Contrary to echoviruses, in coxsackieviruses B1, B3, B5, and A21 in particular, it was found that CD55 supports the binding of these viruses to the cell surface, but does not facilitate entry and lytic infection (Shafren et al., 1995, 1997); the coxsackieviruses B3 and A21 exploit intercellular adhesion molecule 1 (ICAM-1) and CAR along with CD55, respectively, for productive infection (Shafren et al., 1997; Milstone et al., 2005). Dissection of human specificity of coxsackievirus B3 depicted that S104 in the CCP2 domain of human CD55 is critical for imparting the specificity (Pan et al., 2015). Apart from the above viruses, enterovirus 70 has also been documented to use CD55 as a facilitator for virus entry (Karnauchow et al., 1996). It, however, interacts with the elements in CCP1 of CD55 (Karnauchow et al., 1998).

### Complement Subversion by Astroviruses

Astroviruses are small (28–35 nm in diameter), non-enveloped viruses that contain single-stranded RNA genome (∼7 kb) of positive polarity (Shastri et al., 1998). The family is composed of two genera – *Mamastrovirus* and *Avastrovirus*. The HAstV belong to genus *Mamastrovirus* and are responsible for causing viral diarrhea in young children (Madeley and Cosgrove, 1975). A series of studies conducted by Krishna and colleagues provided clear evidence that the coat protein of HAstVs is capable of inhibiting the CP and LP of complement (Sharp et al., 2014).

Human astroviruses are classified into eight serotypes of which serotype 1 is the most predominant worldwide. Early efforts demonstrated that serotypes 1 as well as 2–4 effectively suppress complement activation (Bonaparte et al., 2008). The viral genome is encapsidated by the coat protein encoded by *ORF2* (Willcocks and Carter, 1993; Lewis et al., 1994). Therefore, to understand the suppression of complement by HAstVs, the purified HAstV coat protein (CoPt) was tested for complement inhibitory activity. Like the viral particles, the CoPt also inhibited the activation of complement, in particular, the CP. Examination of the target protein for the CoPt demonstrated that it interacts with the A-chain of C1q (Bonaparte et al., 2008). Further investigations to dissect the mechanism of complement inhibition revealed that binding of CoPt to C1q results in inhibition of C1 activation owing to displacement of the protease tetramer (C1s–C1r–C1r–C1s) (Hair et al., 2010). Since the LP component MBL is structurally and functionally similar to C1q, CoPt was also assessed for its ability to bind to MBL and inhibit the LP. As expected, the protein effectively performed both these functions. The protein though did not bind to the MBL variant Lys55Gln that fails to bind to MASP-2 and activate the LP (Hair et al., 2010). Examination of sequence homology of CoPt with other C1q binding proteins showed limited homology with neutrophil defensin-1, which helped identification of a 30 amino acid peptide of the CoPt that inhibited C1 activation (Gronemus et al., 2010). A smaller yet more potent analog of this peptide was also made which can be developed further for blocking complement-mediated inflammation observed in various clinical conditions (Sharp et al., 2014, 2015).

### Complement Subversion by Togaviruses

Togaviruses are simple, spherical shaped (60–70 nm in diameter), enveloped, positive strand single-stranded RNA viruses with a genome of 9.7–11.8 kb (Fenner et al., 1987). They are divided into two genera namely the Alphavirus and Rubivirus. Among these, the members of the alphaviruses have been shown to interact with complement. For example, complement activation and complement-mediated damage have been reported during Ross River virus infection (Morrison et al., 2007, 2008), while resistance to complement-mediated viral clearance has been reported during Sindbis virus infection (Hirsch et al., 1981).

During the late 1970s and early 1980s when complement was discovered to play an important role during viral infection, Sindbis virus was found to activate the AP (Hirsch et al., 1980a). By this time, it was also known that membrane sialic acid favors interaction with the AP regulator factor H, which in turn prevents the activation of AP on the cell surface. Consequently, it was examined whether sialic acid acquired by Sindbis virus from the host cell during budding affects its ability to activate the AP. As anticipated, the ability of the virus to activate AP was inversely related to its sialic acid content (Hirsch et al., 1981). Moreover, the *in vivo* clearance rate of the virus with less sialic acid content was higher compared to the virus having more sialic acid content and this was dependent on complement (Hirsch et al., 1981). This raised the question – are individuals differentially susceptible to Sindbis virus infection owing to their ability to modify the sialic acid content of the virus? The answer came from the infection experiment performed in outbred Swiss mice. The results demonstrated that tissue sialic acid content of the host influences the ability to resist the infection (Hirsch et al., 1983). Thus, these data supported the proposition that host sialic acid content embodies a way of natural resistance to Sindbis virus infection (Hirsch et al., 1983).

### Complement Subversion by Orthomyxoviruses

The members of the family *Orthomyxoviridae* are helical to spherical enveloped particles ranging from 80 to 120 nm in size (Hale et al., 2010). They contain a negative-sense, single-stranded, segmented RNA genome and are divided into six genera: INFLV type A, B and C, Isavirus, Thogotovirus, and Quaranilvirus. Amongst the INFLVs, type A viruses are most pathogenic and cause significant morbidity and mortality in humans. Influenza viruses have been shown to subvert complement by acquiring CD59 on their envelope (Shaw et al., 2008) and inhibiting C1q mediated recognition of the virions by M1 protein (Zhang et al., 2009). Very recently, recognition of the pandemic influenza A(H1N1) 2009 virus by the AP, in particular, was shown to be hampered due to the inability of human C3b to recognize the viral surface (Rattan et al., 2017).

About a decade ago, Shaw et al. (2008) had demonstrated that like other enveloped viruses (pox, herpes, and retroviruses), INFLV also acquired CD59 on its envelope. Thus, it can be envisaged that the acquired CD59 would protect the virus from the MAC-mediated lysis. However, it should be mentioned here that multiple reports have now established that coating of the INFLV surface with C3b is enough for its neutralization (Jayasekera et al., 2007; Rattan et al., 2017). Therefore, though recruited CD59 would protect it from lysis, it would not shield it from neutralization. Another evasion strategy of INFLV is inhibition of neutralization by blocking the interaction of C1q with antibodies bound to the viral surface (Zhang et al., 2009). Studies on M1 protein revealed that its N-terminal is capable of interacting with the globular region of A-chain of C1q. Further, such interaction also prevented the complement-mediated neutralization of INFLV *in vitro*. What remained unanswered is how M1 interacts with C1q as it underlies the lipid envelope. The authors argue that such interaction is possible when infected host cells are destroyed and M1 protein which is not assembled into the virus particle is released. This premise, however, has not yet been tested.

Activation of the AP of complement is governed by the deposition of C3b molecules onto the target surface. Hence, when C3b deposition onto the target is regulated by surface complement regulators, or when target surface is not amenable to C3b deposition, activation of the AP does not proceed (Sahu et al., 1994; Volanakis and Frank, 1998). Since C3b deposition on INFLV surface results in its neutralization (Jayasekera et al., 2007), we recently compared the susceptibility of the seasonal influenza A(H3N2) virus and pandemic influenza A(H1N1) 2009 virus to AP-mediated neutralization. We observed that unlike the seasonal H3N2 virus, the pandemic H1N1 virus is resistant to neutralization by the AP. Further, activation of C3 near the viral surface showed efficient deposition of C3b onto the seasonal virus, but not on the pandemic INFLV, indicating that pandemic H1N1 virus surface is not amenable to C3b deposition (Rattan et al., 2017). We thus suggest that C3b acceptor site(s) on the pandemic H1N1 virus surface (i.e., on glycoproteins hemagglutinin and neuraminidase) are altered in the pandemic INFLV. Overall, our data suggest that the pandemic INFLV has devised a subtle mechanism to protect itself from the AP-mediated assault.

### Complement Subversion by Paramyxoviruses

Paramyxoviruses are enveloped, single-stranded, negative-sense, non-segmented RNA viruses with a genome of about 15-19 kb (Loney et al., 2009). They are usually spherical and vary in size from 150 to 350 nm. The family *Paramyxoviridae* is divided into two subfamilies: *Paramyxovirinae* and *Pneumovirinae*. The important examples known to subvert complement are MeV, MuV, PIV5, human parainfluenza virus and NiV.

It is known since long that paramyxoviruses are neutralized by complement, particularly by the AP of complement (Hirsch et al., 1986; Devaux et al., 1996; Johnson et al., 2008), and sensitivity to such neutralization varies depending on the cell line through which viruses are passed (Welsh, 1977). In viruses such as PIV5 and MuV, it was observed that C3b attached to the viral surface is predominantly in its inactive form (iC3b form) (Johnson et al., 2008), suggesting the virus may acquire complement regulators from the host cells. Thus, Johnson et al. performed a systematic examination of such incorporation of complement regulators by PIV5 and MuV and dissected their role in limiting the neutralization. They found that both PIV5 and MuV incorporate CD46 which is responsible for the slower rate of their inactivation by the complement system (Johnson et al., 2009). Next, they looked if PIV5 and MuV also acquire CD55 to provide a more robust protection against complement and, of the two, which regulator provides better protection. Their results showed that both the regulators add to protection from complement, but virion-associated CD55 confers better protection than CD46 (Johnson et al., 2012; Li et al., 2016). Apart from the simple acquisition of CD55 by paramyxoviruses, some of the viruses such as PIV5 virus and respiratory syncytial virus were also shown to increase the expression of CD55 on the infected cells (Li et al., 2016).

Unlike the above-mentioned paramyxoviruses, MeV has not been shown to incorporate CD46 or CD55. However, multiple laboratories have identified that CD46 acts as a cellular receptor for the Edmonston strain and all vaccine strains derived from it; the wild-type strains, however, do not use CD46 to gain cellular entry (Dorig et al., 1993; Naniche et al., 1993; Manchester et al., 1994). Interestingly, all the four common isoforms of CD46 (C1, C2, BC1, and BC2) have been shown to function as MeV receptor (Manchester et al., 1994). The MeV contains two major proteins the hemagglutinin (H) and fusion glycoprotein (F), which mediate cell attachment and membrane fusion, respectively. Examination of these proteins for interaction with CD46 indicated that it forms a complex with H, but not F (Nussbaum et al., 1995). Further, characterization of the interaction between MeV and CD46 using CD46/CD55 chimeras and monoclonal antibodies showed that the interaction is mediated only by CCP domains 1 and 2 (Manchester et al., 1997).

Nipah virus is a zoonotic virus that causes highly fatal (38–75%) febrile human encephalitis (Rockx et al., 2012). Importantly, human-to-human transmission of NiV has also been documented, which has increased the concern (Gurley et al., 2007). During the study of complement-mediated neutralization of pseudotyped particles containing NiV glycoproteins F and G, it was noted that the pseudotyped particles efficiently activate the AP, but unlike other paramyxoviruses, show resistance to neutralization; the particles, however, were susceptible to neutralization by the CP (Johnson et al., 2011). To determine the basis of this resistance, the authors extended this work with bonafide NiV particles (Johnson et al., 2015). *In vitro* functional assays performed using purified NiV and complement proteins showed that NiV possesses a factor I-like protease activity that inactivates C3b in the presence of factor H and CD35, but not CD46. Moreover, the NiV factor I-like protease did not inactivate C4b when incubated with CD35 and C4b-binding protein suggesting the viral protease activity differs from that of human factor I to some extent. Electron microscopy of the purified NiV showed labeling of the surface with anti-factor I. Given that NiV surface glycoproteins F and G do not have any homology to human factor I, the most likely possibility is that one or both of these proteins interact with factor I to recruit it on NiV surface.

## Concluding Remarks

The presence of the complement system is not restricted to blood rather it is ubiquitous, including in the immune privileged sites – the brain and eye. Thus, viruses are vulnerable to complement assault in the blood as well as at the various tissue sites. It is clear from the diverse examples discussed here that to be a successful pathogen, largely viruses either acquire or encode proteins that help them subvert the complement system. The salient points that emerge are: (i) viruses target all the three major pathways of complement suggesting that all the pathways participate in controlling viral infections, (ii) large DNA viruses encode their own homologs of the cellular complement regulators. This, however, is limited only to poxviruses and gamma herpesviruses. Whether this is related to their ability to establish latent infection as proposed (Chen et al., 2016), however, is not yet clear as other viruses which are capable of causing latent infection are not known to encode homologs of the cellular complement regulators, (iii) cellular homologs encoded by viruses function at high nanomolar concentrations, which are expected to be achieved only at the site of infection. These data thus point out that local complement plays a critical role in containing viral infections, (iv) there exists a built-in redundancy for complement regulation in viruses denoting an essential role of complement in controlling viruses, and (v) enveloped viruses deftly acquire membrane complement regulatory proteins such as CD55, CD46, and CD59. Since these proteins function in a species-specific manner or exhibit ‘homologous restriction,’ incorporation of these in virions eliminate the need to encode a regulator that would work in multiple susceptible species. This, however, does not mean that virally encoded regulators do not accumulate adaptive mutations to increase viral fitness in the new host environment. A critical example of this is the poxviral complement regulators wherein mutations in VCP and SPICE have been shown to skew their specificity toward their respective host complement (Yadav et al., 2012). Such common evasion mechanism though was not found in non-enveloped viruses.

Up until recently, it was thought that complement activation occurs only in the extracellular milieu and hence, the system is capable of opsonizing viruses only outside the cell. It is, however, now clear that the major complement proteins like C3 and C5 can be cleaved inside the cell in a non-canonical way (Liszewski et al., 2013; Arbore et al., 2016). This implies that opsonization of viruses with C3b may happen even inside the cell. Tam et al. (2014) have elegantly demonstrated that opsonization of viral particles with C3b outside the cell and sensing of these C3b coated viral particles inside the cell trigger mitochondrial antiviral signaling (MAVS)-dependent immune signaling as well as proteasome-mediated viral degradation resulting in restriction of viral infection. It is, therefore, likely that opsonization of viral particles with C3b inside the cells may have the similar fate. Further, it has also been documented that cleavage of C3 and C5 inside the T cell and engagement of the respective receptors for their cleaved products in an autocrine fashion dictate T_H_1 induction (Yamamoto et al., 2013). Consequently, the intracellularly generated C3a and C5a are expected to play a predominant role in generating efficient protective immune response against viruses. This raises the question – how do T cell tropic viruses manipulate the non-canonical complement activation inside the cell for their survival? And more importantly, in general, how viruses regulate intracellular complement activation? A better understanding of this would not only add to our knowledge of yet unknown immune evasion mechanisms devised by viruses, but would also aid in rational vaccine design that still remains a major challenge.

## Author Contributions

PA, RN, HO, and AS conceived the concept for this review article. PA, RN, HO, JK, and AS wrote the manuscript. PA, RN, HO, JK, and AS read, edited, and reviewed the manuscript.

## Conflict of Interest Statement

The authors declare that the research was conducted in the absence of any commercial or financial relationships that could be construed as a potential conflict of interest.

## Abbreviations

AdV: adenoviruses
AP: alternative pathway
CAR: coxsackie and adenovirus receptor
CCP: complement control protein
CoPt: human astrovirus coat protein
CP: classical pathway
CR: complement receptor
DAF; CD55: decay-acceleration factor
DENV: dengue virus
EBV: Epstein-Barr virus
EV: ectromelia virus
gC: glycoprotein C
HAstV: human astrovirus
HCMV: human cytomegalovirus
HCV: hepatitis C virus
HHV: human herpesvirus
HIV-1: human immunodeficiency virus-1
HSV: herpes simplex virus
HVS: Herpesvirus saimiri
HTLV-1: human T-cell leukemia virus type 1
INFLV: influenza virus
JEV: Japanese encephalitis virus
KSHV, HHV-8: Kaposi’s sarcoma-associated herpesvirus
γ-HV68: murine gammaherpesvirus 68
LP: lectin pathway
MAC: membrane attack complex
MBL: mannose-binding lectin
MCMV: murine cytomegalovirus
MCP; CD46: membrane cofactor protein
MeV: measles virus
MPXV: monkeypox viruses
MuV: mumps virus
NiV: Nipah virus
NS1: non-structural protein 1
PIV5: parainfluenza virus Type 5
RCA: regulators of complement activation
RRV: rhesus rhadinovirus
SAMHD1: SAM domain and HD domain-containing protein 1
SARS: severe acute respiratory syndrome
SIV: simian immunodeficiency virus
SPICE: smallpox inhibitor of complement enzymes
VACV: vaccinia virus
VCP: vaccinia virus complement control protein
VSV: vesicular stomatitis virus
WNV: West Nile virus
YFV: Yellow fever virus
ZIKV: Zika virus
